# A Patient Safety Educational Tool for Patients With Chronic Kidney Disease: Development and Usability Study

**DOI:** 10.2196/16137

**Published:** 2020-05-28

**Authors:** Cassandra Bowman, Joseph Lunyera, Aviel Alkon, L Ebony Boulware, Jennifer St Clair Russell, Jennie Riley, Jeffrey C Fink, Clarissa Diamantidis

**Affiliations:** 1 Division of General Internal Medicine Duke University School of Medicine Durham, NC United States; 2 The National Kidney Foundation Washington, DC United States; 3 Division of General Internal Medicine University of Maryland School of Medicine Baltimore, MD United States; 4 Division of Nephrology Duke University School of Medicine Durham, NC United States; 5 Duke Department of Population Health Sciences Duke University School of Medicine Durham, NC United States

**Keywords:** patient safety, chronic kidney disease, patient education, mhealth

## Abstract

**Background:**

Chronic kidney disease (CKD) is a health condition that threatens patient safety; however, few interventions provide patient-centered education about kidney-specific safety hazards.

**Objective:**

We sought to develop and test the usability of a mobile tablet–based educational tool designed to promote patient awareness of relevant safety topics in CKD.

**Methods:**

We used plain language principles to develop content for the educational tool, targeting four patient-actionable safety objectives that are relevant for individuals with CKD. These four objectives included avoidance of nonsteroidal anti-inflammatory drugs (NSAIDs); hypoglycemia awareness (among individuals with diabetes); temporary cessation of certain medications during acute volume depletion to prevent acute kidney injury (ie, “sick day protocol”); and contrast dye risk awareness. Our teaching strategies optimized human-computer interaction and content retention using audio, animation, and clinical vignettes to reinforce themes. For example, using a vignette of a patient with CKD with pain and pictures of common NSAIDs, participants were asked “Which of the following pain medicines are safe for Mr. Smith to take for his belly pain?” Assessment methods consisted of preknowledge and postknowledge surveys, with provision of correct responses and explanations. Usability testing of the tablet-based tool was performed among 12 patients with any stage of CKD, and program tasks were rated upon completion as no error, noncritical error (self-corrected), or critical error (needing assistance).

**Results:**

The 12 participants in this usability study were predominantly 65 years of age or older (n=7, 58%) and female (n=7, 58%); all participants owned a mobile device and used it daily. Among the 725 total tasks that the participants completed, there were 31 noncritical errors (4.3%) and 15 critical errors (2.1%); 1 participant accounted for 30 of the total errors. Of the 12 participants, 10 (83%) easily completed 90% or more of their tasks. Most participants rated the use of the tablet as very easy (n=7, 58%), the activity length as “just right” (rather than too long or too short) (n=10, 83%), and the use of clinical vignettes as helpful (n=10, 83%); all participants stated that they would recommend this activity to others. The median rating of the activity was 8 on a scale of 1 to 10 (where 10 is best). We incorporated all participant recommendations into the final version of the educational tool.

**Conclusions:**

A tablet-based patient safety educational tool is acceptable and usable by individuals with CKD. Future studies leveraging iterations of this educational tool will explore its impact on health outcomes in this high-risk population.

## Introduction

Chronic kidney disease (CKD) is characterized by progressive loss of kidney function and increases the risk of adverse patient safety events [1]. As kidney function declines, renal clearance is reduced, which can result in elevated and potentially hazardous circulating levels of medications. For example, individuals with CKD and concomitant diabetes who are on antidiabetic medications are at heightened risk of developing hypoglycemia due to the delayed renal clearance of these therapies [2]. Furthermore, as CKD progresses, susceptibility to hemodynamic changes or nephrotoxins such as nonsteroidal anti-inflammatory drugs (NSAIDs) is amplified, significantly increasing the risk of acute kidney injury (AKI) development and accelerated renal function decline [3,4].

Despite these well-established risks, patient awareness of CKD and of its potential safety hazards remains low, which limits the effectiveness of current CKD treatment strategies [5,6]. A high prevalence of limited health literacy among individuals with CKD [7] and poor patient-provider communication about CKD and its risk factors all contribute to low awareness and ineffective patient self-management among individuals with kidney disease [8]. Consequently, behaviors linked with CKD risk and progression, such as NSAID use, are commonly reported by individuals at high risk of adverse outcomes [9]; however, few interventions have been developed to address these challenges and attenuate risks.

We hypothesized that educational tools tailored to audiences with low health literacy can increase disease awareness and improve self-management among individuals with kidney disease. Therefore, we sought to develop a digital educational tool to promote patient awareness of relevant patient safety issues in CKD. Here, we describe the development of this educational tool; our experience usability testing its digital platform among the target patient population; and the results of our assessment of the users’ experience with the educational tool.

## Methods

### Educational Tool Development

This tablet-based educational tool was developed to support patient awareness of important disease-specific patient safety topics relevant for individuals with CKD. The tool was designed to evaluate participants’ awareness of common safety risks associated with reduced kidney function and to provide education regarding optimal patient-centered behavior to minimize risk. Materials were designed for low health literacy audiences, as it is estimated that the health literacy of approximately 25% of the general CKD population is inadequate [10]. The educational content was developed using Adult Learning Theory and plain language principles [11]. The andragogy theory of adult learning by Malcolm Knowles (Adult Learning Theory) postulates that adult learning is motivated by 6 principles: (1) the need to know, or “why” a person should learn; (2) a foundation or experience that provides the basis for learning activities; (3) a self-concept, or the idea that adults need to be responsible for and take charge of their learning; (4) readiness to learn stemming from perceived relevance of the knowledge to be gained; (5) orientation, or the concept that adults learn best with task-oriented learning that exercises their problem-solving ability; and (6) internal motivation [11]. The educational content of the tool draws on this framework to promote CKD awareness and self-management.

The educational objectives of the tool were based on patient safety topics relevant to individuals with reduced renal function based on prior work examining adverse safety events in CKD [1,12-14]. Educational content was created using previously derived educational materials addressing patient safety in CKD [15]; this content was reviewed and refined by adult educational curriculum experts using a rubric based on Adult Learning Theory [16]. The educational material encompassed four patient safety objectives pertaining to CKD: (1) avoidance of NSAIDs [17,18], (2) hypoglycemia awareness (among individuals with diabetes) [19], (3) temporary cessation of certain medications during acute volume depletion (ie, “sick day protocol”) [5,20,21], and (4) iodinated contrast risk awareness [22-24] (Table 1).

**Figure 1:**
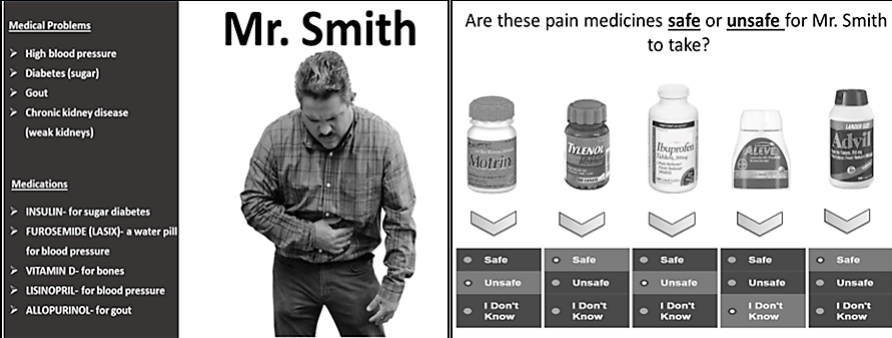
Example of an educational tool vignette.

The developed teaching strategies optimized human-computer interaction and content retention. The hypothetical experiences of 2 diverse patients (Mr. Smith and Mrs. Johnson), with Hispanic and African American backgrounds, respectively, were used to provide clinical context (Figure 1). Simple animations accompanied the audio presentations in real time and emphasized key concepts [11,25]. Visual images and audio were chosen in consideration of information clarity, participant involvement, and captions to meet participant comprehension needs [26,27] (see Multimedia Appendices 1 and 2).

**Table 1:** Patient safety objectives, aims, instructional content, teaching strategies, and assessment methods of the developed tool.

Patient safety objective and aim		Instructional content	Teaching strategies	Assessment methods
**NSAIDs^a^**				
	To distinguish safe pain medications for individuals with CKD^b^ from unsafe ones	Description of common pain medications available over the counter, their common names/brands, and their safety for use by individuals with CKD	Auditory explanations with complimentary graphics that include photographs of common brands of pain medication and their safety for CKD, linked with a clinical scenario and questions regarding a hypothetical patient experiencing pain	Clinical scenario of a second hypothetical patient experiencing pain, interactive questions that use photographs of common brands of pain medications and ask the learner to distinguish between safe and unsafe pain medications
“**Sick day protocol”**				
	To be aware of the symptoms of volume depletion and the potential danger associated with taking certain medications during volume depletion	Description of common scenarios linked with volume depletion (eg, diarrhea, vomiting, fever); list of different medications that can harm the kidneys if taken during volume depletion (eg, diuretics and ACE^c^ inhibitors)	Auditory explanations with complimentary graphics that include photographs of medications to be withheld during volume depletion, linked with a clinical scenario and questions regarding a hypothetical patient experiencing volume depletion	Clinical scenario of a second hypothetical patient with volume depletion and interactive questions that ask the learner to choose which medications should be withheld during a “sick day”
**Hypoglycemia**				
	To understand the concept of hypoglycemia and the elevated risk of hypoglycemia in individuals with CKD	Description of the link between CKD and diabetes, how diabetic therapies can lead to hypoglycemia, and common symptoms of hypoglycemia	Auditory explanations with complimentary graphics that include photographs of a glucometer with a low blood sugar reading, linked with a clinical scenario and questions regarding a hypothetical patient experiencing signs and symptoms of hypoglycemia	Clinical scenario of a second hypothetical patient with hypoglycemia and interactive questions that ask the learner to choose medications that could lead to hypoglycemia and steps that should be taken if the patient suspects they have hypoglycemia
**Contrast dyes**				
	To understand how medical tests that use contrast dyes can further harm already weak kidneys	Description of common medical tests that use contrast dyes and emphasis on the importance of informing health care providers about their CKD	Auditory explanations with complimentary graphics that include descriptions of common medical tests, linked with a clinical scenario and questions regarding a hypothetical patient undergoing a medical test	Clinical scenario of a second hypothetical patient undergoing a medical test and interactive questions that ask the learner to distinguish between safe and unsafe medical tests for individuals with CKD

^a^NSAIDs: nonsteroidal anti-inflammatory drugs.

^b^CKD: chronic kidney disease.

^c^ACE: angiotensin-converting enzyme.

### Usability Testing

We recruited 12 patients receiving care from outpatient CKD clinics at Duke University Hospital in fall 2016 and again in early 2018. Individuals who were non–English-speaking, illiterate, physically unable to use a tablet (ie, individuals with limb amputation, debilitating arthritis, or legal blindness), on dialysis, or under the age of 18 years were excluded. If interested, patients were taken to a private room, where written consent was obtained and testing was initiated. The study was approved by the Institutional Review Board of Duke University. All participants were compensated for their time.

### Testing Protocol

The usability testing was based on the performance of the users and their ability to answer questions and navigate the educational tool on a tablet device. Participants were given a pair of headphones connected to an Android tablet (Samsung Galaxy Tab A, model SM-T550). Once seated comfortably, participants were asked to complete a series of tasks designed to evaluate their ability to interact with the technical aspect of the safety tool (Multimedia Appendix 3). All participants were asked to complete a minimum of 52 tasks and a maximum of 67 tasks based on their diabetes status. The study facilitator remained in the room with the participant and monitored the ease or difficulty with which tasks were completed as well as participant commentary about the activity. Task completions were categorized as no error, noncritical error, or critical error. If the participant had no difficulty completing the task independently, the task was marked as no error (easily completed). A noncritical error was defined as a task that was completed with an alternative strategy. For example, if a participant chose a wrong answer but made the correction before selecting “check my answers,” the error was categorized as noncritical. A critical error was defined as a task that could not be completed independently by the participant without assistance from study personnel. After completing the tool, participants were asked to evaluate their user experience with the tool.

### Statistical Analysis

No formal hypothesis testing was performed in this analysis due to the qualitative study methods of usability testing. Participants’ demographic characteristics and their typical use of the internet and mobile devices were described using counts and percentages. Participant satisfaction using the educational tool and the tablet was recorded. Critical and noncritical errors were evaluated for each subject along with the time to complete each exercise. Errors and completion times were analyzed to recognize any significant differences between participants who regularly use the internet and mobile apps and participants who do not.

## Results

### Usability Testing

Twelve participants took part in the usability testing of the patient safety educational tool. The majority of participants were aged 45 years and older (11/12, 92%) and were female (7/12, 58%) (Table 2). The races of the participants were diverse. All participants had at least completed high school and owned a mobile device that they used daily. Half the population owned a tablet they used for internet and mobile apps. Most participants used mobile apps (9/12, 75%) and the internet (8/12, 67%).

**Figure 2:**
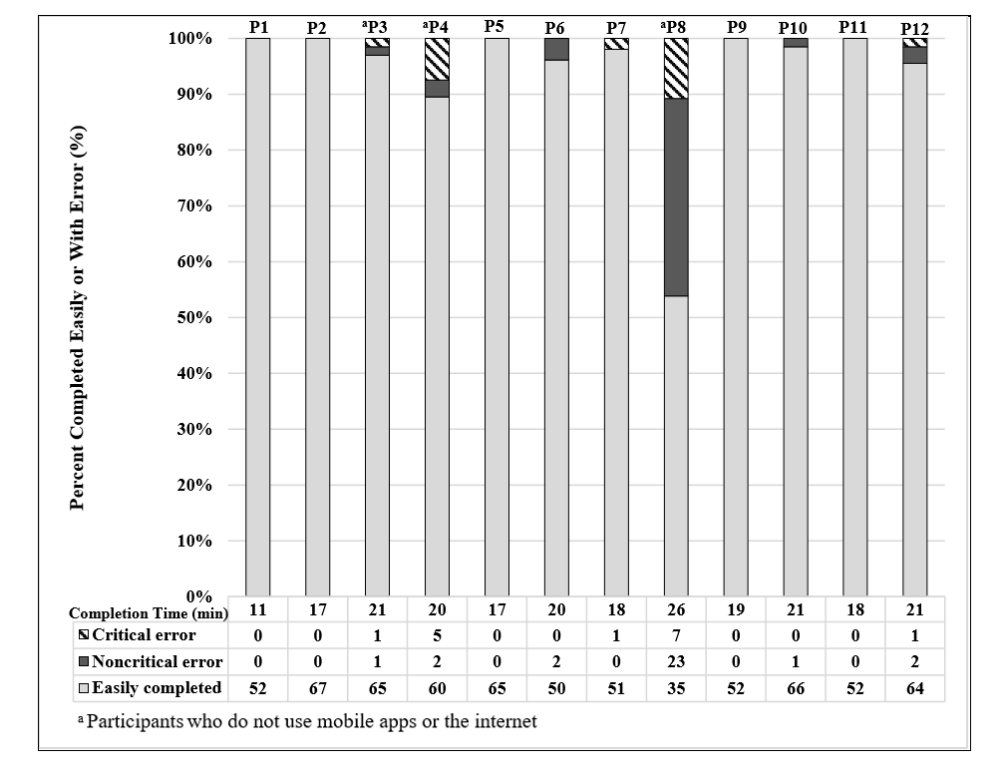
Usability testing results by participant.

Figure 2 illustrates user performance by displaying the numbers of critical errors, noncritical errors, and easily completed tasks out of the total assigned tasks. Out of 725 tasks, 15 critical errors (2.1%), 31 noncritical errors (4.3%), and 679 easily completed tasks (93.7%) were recorded. Of the 12 participants, 10 (83%) easily completed at least 90% of their tasks. The median time to complete all tasks was 19 minutes (SD 3.4 minutes, range 11-26 minutes). The median time for internet users to complete all tasks was 18 minutes (range 11-21 minutes); in contrast, participants who did not use the internet completed all tasks with a median time of 21 minutes (range 20-26 minutes). There was 1 outlier who completed all tasks in 11 minutes. The participants who used the internet had fewer errors than those who did not: 4/725 errors versus 42/725 (0.55% versus 5.8%, respectively). The 3 participants who did not use the internet or mobile apps were all aged 65 years or older. Five participants completed the testing without any errors, and 2 others completed it with only 1 error. Participants 4 and 8 had the most difficulty, with 7 and 30 errors, respectively. Both of these participants were over the age of 65, did not own a tablet, did not use the internet, and did not use mobile apps. All subjects completed more than 50% of the exercises without any errors, and 11/12 subjects (92%) completed more than 90% of the tasks without critical errors.

**Table 2:** Usability study participant demographics (N=12).

Characteristic		n (%)
**Age (years)**		
	<45	1 (8)
	45-64	4 (33)
	≥65	7 (58)
**Gender**		
	Male	5 (42)
	Female	7 (58)
**Race**		
	Black/non-Hispanic	6 (50)
	White/non-Hispanic	5 (42)
	Hispanic	1 (8)
**Education**		
	High school (grades 9-12) or GED^a^	3 (25)
	Some college but did not graduate	4 (33)
	College	1 (8)
	Graduate or professional school	4 (33)
**Mobile devices used**		
	Cell phone	12 (100)
	Tablet	6 (50)
**Frequency of mobile device use**		
	Daily	12 (100)
**Type of mobile device use**		
	Apps	9 (75)
	Internet	8 (67)

^a^GED: General Educational Development

### User Experience Survey

The Likert scale used to evaluate user satisfaction using the tablet application is shown in Table 3. Participants were asked questions about their experience using the app and provided a ranking for the activity from 0-2 or 0-4, with lower numbers indicating higher satisfaction. The median and range for the ranking of the tool were used to determine the results. The median ranking for the overall activity was 0 (range 0-4), which describes the activity as “very easy.” Participants found the tablet to be very easy to use based on the median ranking of 0 (range 0-4). The length of time to complete the activity had a median ranking of 1 (range 0-2), indicating that the length was “just right.” The subjects ranked the patient story examples and audio as 1 (range 0-4), stating they agree that these aspects of the app helped them to understand the information. When participants were asked if they would recommend the activity to others, the median ranking was 1 (range 0-4), indicating that the participants agree they would recommend the app to others. Lastly, the participants were asked how they would rate the activity on a scale of 1-10 (10 being the best); they provided a median rating of 8 (range 0-10).

After completion of the activity, participants were given the opportunity to provide feedback and comments about the tool, such as what changes they would make, what they liked or did not like, and their overall thoughts about the exercise. Most comments reiterated the helpfulness and quality of the material learned using the app. Other comments suggested ways to improve the tool by stating that the exercise should be faster to navigate, while others expressed the need to simplify some medication names. The majority of participants found the educational tool to be a worthwhile experience that helped them learn about adverse safety events (Multimedia Appendix 4).

**Table 3:** Survey indicating user satisfaction with the patient safety tool.

Question	Response, median (range)
Overall, this activity was…^a^	0 (0-4)
The mobile tablet was…^a^	0 (0-4)
The length of time it took to complete the activity was…^b^	1 (0-2)
Using stories about patients helped me to understand the information.^c^	1 (0-4)
The use of audio helped me to understand the information.^c^	1 (0-4)
I would recommend this activity to others.^c^	1 (0-4)
How would you rate this activity?^d^	8 (0-10)

^a^Possible responses: 0=Very easy, 1=Somewhat easy, 2=Neither easy nor difficult, 3=Somewhat difficult, 4=Very difficult.

^b^Possible responses: 0=Too long, 1=Just right, 2=Too short.

^c^Possible responses: 0=Strongly agree, 1=Agree, 2=Neutral, 3=Disagree, 4=Strongly disagree.

^d^Possible responses: 1-10, where 1 is the worst and 10 is the best.

## Discussion

The CKD educational tool was designed to help audiences with low health literacy learn about potential safety hazards associated with kidney disease through an interactive and simple-to-use digital interface. Our findings demonstrate that the majority of participants found the digital tool to be helpful and easy to navigate and would recommend it to others. Most participants were proficient in using the tablet without significant guidance and without errors; those with critical errors were older than 65 years and less familiar with digital technologies in general but still predominantly completed the programming without difficulty. The participants’ ability to complete the tasks did not appear to be related to their degrees of education, which supports our intent to provide an educational tool for populations with low health literacy regardless of educational background. Although some participants did not regularly use the internet or mobile apps, the task completion times in this group did not greatly differ from those of regular internet users. Together, these findings suggest that further testing to evaluate the impact of the CKD educational tool on outcomes is warranted.

CKD places individuals at high risk for developing adverse safety events, and effective tools that attenuate this heightened risk are needed. The principal feature of CKD that can influence patient risk is the reduced level of renal function or glomerular filtration rate (GFR), which is typical of the condition. A low GFR affects the clearance of many drugs, confounds therapeutic interventions, and increases susceptibility to acute kidney injury [28]. Moreover, CKD is frequently associated with several comorbidities, such as diabetes and cardiovascular disease [28]. The clustering of these health conditions leads to increased inpatient and outpatient medical encounters, with potential consequences of polypharmacy and high self-management burden. Compounded by low patient awareness of kidney disease [29] and poor knowledge of contributory health behaviors [5,30], potential adverse outcomes in CKD are abundant.

Our previous work demonstrated high acceptance of digital tools and general ease of use among individuals with CKD, supporting mobile health (mHealth) as a feasible mechanism through which patient-centered programming can be delivered to CKD populations [31-33]. Other studies using mHealth tools in non-CKD populations have demonstrated similar results for provision of tailored educational programming across an array of health conditions, such as obesity, asthma, and diabetes [34-37]. For individuals with CKD, responsiveness to the high degree of limited health literacy in the CKD population [10,38] is fundamental to the development of any educational curriculum, and use of audio and visual support can address differences in learning styles. We developed our app using plain language principles to address this need. Similarly, user experience testing of such digital tools in the CKD population can unearth interface difficulties by users and inform refinements to optimize user understanding and retention. The importance of such assessment is evidenced by one recent study that systematically evaluated all available and updated patient-facing CKD mobile apps. Of the 174 unique applications found, only 38 were related to kidney disease, were patient-facing, and had been updated in the previous 4 years. The quality of app content widely varied, and the authors found high levels of discordance between patient and provider app reviewers regarding value and usability [39]. These findings highlight the need for digital health content that is developed with a patient-centric approach and tested for ease of use in the target population *prior* to implementation in clinical care and broad dissemination. Similar content should also be made available through alternative platforms, such as on paper or via a website, to address differences in digital readiness and access.

Empowering patients with proper information and education in a manner they can comprehend can improve self-efficacy and self-management and, in turn, improve health outcomes [40]. The patient-actionable tool we developed emphasizes the importance of a patient-centered approach to educating CKD patients about self-care at home. Further, developing digital tools for patient education can bridge the digital gap between younger and older generations. When provided with online tools to manage chronic illnesses, older individuals access the digital content more frequently and sustain its use over longer periods than their younger counterparts [41]. This engagement by older populations is particularly relevant in CKD, as its onset typically occurs later in life. Digital tools can also narrow disparities in accessibility between rural and urban CKD populations, especially with the widespread availability of smartphones and the mobile web as well as the increasing use of telehealth applications for clinical care [42,43]. However, in kidney disease, few tools have been appropriately developed for use in a low health literacy population or have undergone formal usability testing. Fewer still tools are available outside of the research setting, which creates opportunities for such tools to be integrated into clinical care and evaluated in pragmatic studies [44,45].

Our study has limitations that are worthy of mention. Although our sample size is within the recommended range of 5-7 participants for usability testing [46,47], our sample size is small, and we recognize that this limits the inferences was can draw from our findings. Further, our participants were recruited from the Duke University Hospital nephrology clinics and may not be representative of the general CKD population, particularly given that only half our study participants were elderly. We also restricted our usability testing to individuals with CKD, which may not be generalizable to individuals with other kidney-related (eg, acute kidney injury) or non–kidney-related conditions. Finally, the specific patient safety conditions included in our educational curriculum may not be comprehensive, although they do represent many common patient safety events reported and detected in patients with kidney disease [1,48].

In summary, usability testing of a patient-centered digital tool to promote safety among patients with CKD demonstrated general ease of use and acceptability. The impact of the intervention on mitigation of safety events in a CKD population has yet to be determined. However, the information derived from this study strengthens the growing consensus that tailored digital tools can be used effectively by high risk populations, including older persons and persons with low health literacy. Future studies are needed to evaluate the benefit of such tools in clinical care.

## Appendix

Multimedia Appendix 1 Screenshots of the app.

Multimedia Appendix 2 Transcript of the app audio.

Multimedia Appendix 3 Task descriptions.

Multimedia Appendix 4 Participant testimonials about using the app.

## Abbreviations

AKI: acute kidney injury
CKD: chronic kidney disease
mHealth: mobile health
NSAIDs: nonsteroidal anti-inflammatory drugs

## References

[1] Ginsberg JS, Zhan M, Diamantidis CJ, Woods C, Chen J, Fink JC (2014). Patient-reported and actionable safety events in CKD. J Am Soc Nephrol.

[2] Moen MF, Zhan M, Hsu VD, Walker LD, Einhorn LM, Seliger SL, Fink JC (2009). Frequency of hypoglycemia and its significance in chronic kidney disease. Clin J Am Soc Nephrol.

[3] He L, Wei Q, Liu J, Yi M, Liu Y, Liu H, Sun L, Peng Y, Liu F, Venkatachalam MA, Dong Z (2017). AKI on CKD: heightened injury, suppressed repair, and the underlying mechanisms. Kidney Int.

[4] Chawla LS, Eggers PW, Star RA, Kimmel PL (2014). Acute kidney injury and chronic kidney disease as interconnected syndromes. N Engl J Med.

[5] Doerfler RM, Diamantidis CJ, Wagner L, Scism BM, Vaughn-Cooke M, Fink WJ, Blakeman T, Fink JC (2019). Usability Testing of a Sick-Day Protocol in CKD. Clin J Am Soc Nephrol.

[6] Plantinga LC, Tuot DS, Powe NR (2010). Awareness of chronic kidney disease among patients and providers. Adv Chronic Kidney Dis.

[7] Taylor DM, Fraser SDS, Bradley JA, Bradley C, Draper H, Metcalfe W, Oniscu GC, Tomson CRV, Ravanan R, Roderick PJ, ATTOM investigators (2017). A Systematic Review of the Prevalence and Associations of Limited Health Literacy in CKD. Clin J Am Soc Nephrol.

[8] Greer RC, Crews DC, Boulware LE (2012). Challenges perceived by primary care providers to educating patients about chronic kidney disease. J Ren Care.

[9] Zhan M, St Peter WL, Doerfler RM, Woods CM, Blumenthal JB, Diamantidis CJ, Hsu C, Lash JP, Lustigova E, Mahone EB, Ojo AO, Slaven A, Strauss L, Taliercio JJ, Winkelmayer WC, Xie D, Fink JC, Chronic Renal Insufficiency Cohort (CRIC) Study Investigators (2017). Patterns of NSAIDs Use and Their Association with Other Analgesic Use in CKD. Clin J Am Soc Nephrol.

[10] Taylor DM, Fraser S, Dudley C, Oniscu GC, Tomson C, Ravanan R, Roderick P, ATTOM investigators (2018). Health literacy and patient outcomes in chronic kidney disease: a systematic review. Nephrol Dial Transplant.

[11] Smith M the encyclopedia of informal education.

[12] Diamantidis CJ, Zuckerman M, Fink W, Hu P, Yang S, Fink JC (2012). Usability of a CKD educational website targeted to patients and their family members. Clin J Am Soc Nephrol.

[13] Wagner L, Tata AL, Fink JC (2015). Patient safety issues in CKD: core curriculum 2015. Am J Kidney Dis.

[14] Chapin E, Zhan M, Hsu VD, Seliger SL, Walker LD, Fink JC (2010). Adverse safety events in chronic kidney disease: the frequency of. Clin J Am Soc Nephrol.

[15] Diamantidis CJ, Fink W, Yang S, Zuckerman MR, Ginsberg J, Hu P, Xiao Y, Fink JC (2013). Directed use of the internet for health information by patients with chronic kidney disease: prospective cohort study. J Med Internet Res.

[16] Knowles M (1985). San Francisco.

[17] Plantinga L, Grubbs V, Sarkar U, Hsu C, Hedgeman E, Robinson B, Saran R, Geiss L, Burrows NR, Eberhardt M, Powe N (2011). Nonsteroidal anti-inflammatory drug use among persons with chronic kidney disease in the United States. Ann Fam Med.

[18] Jang SM, Cerulli J, Grabe DW, Fox C, Vassalotti JA, Prokopienko AJ, Pai AB (2014). NSAID-avoidance education in community pharmacies for patients at high risk for acute kidney injury, upstate New York, 2011. Prev Chronic Dis.

[19] Gianchandani RY, Neupane S, Iyengar JJ, Heung M (2017). PATHOPHYSIOLOGY AND MANAGEMENT OF HYPOGLYCEMIAIN END-STAGE RENAL DISEASE PATIENTS: A REVIEW. Endocr Pract.

[20] MacCallum L, Senior PA (2019). Safe Use of Metformin in Adults With Type 2 Diabetes and Chronic Kidney Disease: Lower Dosages and Sick-Day Education Are Essential. Can J Diabetes.

[21] Morris RL, Ashcroft D, Phipps D, Bower P, O'Donoghue D, Roderick P, Harding S, Lewington A, Blakeman T (2016). Preventing Acute Kidney Injury: a qualitative study exploring 'sick day rules' implementation in primary care. BMC Fam Pract.

[22] Ogata N, Ikari Y, Nanasato M, Okutsu M, Kametani R, Abe M, Uehara Y, Sumitsuji S (2014). Safety margin of minimized contrast volume during percutaneous coronary intervention in patients with chronic kidney disease. Cardiovasc Interv Ther.

[23] Gandhi S, Mosleh W, Abdel-Qadir H, Farkouh ME (2014). Statins and contrast-induced acute kidney injury with coronary angiography. Am J Med.

[24] Vanmassenhove J, Kielstein J, Jörres A, Biesen WV (2017). Management of patients at risk of acute kidney injury. Lancet.

[25] David TJ, Patel L (1995). Adult learning theory, problem based learning, and paediatrics. Archives of Disease in Childhood.

[26] Morony S, McCaffery KJ, Kirkendall S, Jansen J, Webster AC (2017). Health Literacy Demand of Printed Lifestyle Patient Information Materials Aimed at People With Chronic Kidney Disease: Are Materials Easy to Understand and Act On and Do They Use Meaningful Visual Aids?. Journal of Health Communication.

[27] Morony S, Flynn M, McCaffery KJ, Jansen J, Webster AC (2015). Readability of Written Materials for CKD Patients: A Systematic Review. Am J Kidney Dis.

[28] Weir MR, Fink JC (2014). Safety of medical therapy in patients with chronic kidney disease and end-stage renal disease. Curr Opin Nephrol Hypertens.

[29] USRDS annual data report.

[30] Siew ED, Parr SK, Wild MG, Levea S, Mehta KG, Umeukeje EM, Silver SA, Ikizler TA, Cavanaugh KL (2019). Kidney Disease Awareness and Knowledge among Survivors of Acute Kidney Injury. Am J Nephrol.

[31] Diamantidis CJ, Ginsberg JS, Yoffe M, Lucas L, Prakash D, Aggarwal S, Fink W, Becker S, Fink JC (2015). Remote Usability Testing and Satisfaction with a Mobile Health Medication Inquiry System in CKD. Clin J Am Soc Nephrol.

[32] Diamantidis CJ, Becker S (2014). Health information technology (IT) to improve the care of patients with chronic kidney disease (CKD). BMC Nephrol.

[33] Tuot DS, Boulware LE (2017). Telehealth Applications to Enhance CKD Knowledge and Awareness Among Patients and Providers. Adv Chronic Kidney Dis.

[34] Shaw RJ, Bosworth HB, Hess JC, Silva SG, Lipkus IM, Davis LL, Johnson CM (2013). Development of a Theoretically Driven mHealth Text Messaging Application for Sustaining Recent Weight Loss. JMIR Mhealth Uhealth.

[35] Lin P, Grambow S, Intille S, Gallis JA, Lazenka T, Bosworth H, Voils CL, Bennett GG, Batch B, Allen J, Corsino L, Tyson C, Svetkey L (2018). The Association Between Engagement and Weight Loss Through Personal Coaching and Cell Phone Interventions in Young Adults: Randomized Controlled Trial. JMIR Mhealth Uhealth.

[36] Pfammatter A, Spring B, Saligram N, Davé R, Gowda A, Blais L, Arora M, Ranjani H, Ganda O, Hedeker D, Reddy S, Ramalingam S (2016). mHealth Intervention to Improve Diabetes Risk Behaviors in India: A Prospective, Parallel Group Cohort Study. J Med Internet Res.

[37] Sage A, Roberts C, Geryk L, Sleath B, Tate D, Carpenter D (2017). A Self-Regulation Theory-Based Asthma Management Mobile App for Adolescents: A Usability Assessment. JMIR Hum Factors.

[38] Taylor DM, Bradley JA, Bradley C, Draper H, Dudley C, Fogarty D, Fraser S, Johnson R, Leydon GM, Metcalfe W, Oniscu GC, Robb M, Tomson C, Watson CJE, Ravanan R, Roderick P, ATTOM investigators (2019). Limited health literacy is associated with reduced access to kidney transplantation. Kidney Int.

[39] Singh K, Diamantidis CJ, Ramani S, Bhavsar NA, Mara P, Warner J, Rodriguez J, Wang T, Wright-Nunes J (2019). Patients' and Nephrologists' Evaluation of Patient-Facing Smartphone Apps for CKD. Clin J Am Soc Nephrol.

[40] Timmers T, Janssen L, Pronk Y, van der Zwaard BC, Koëter S, van Oostveen D, de Boer S, Kremers K, Rutten S, Das D, van Geenen RC, Koenraadt KL, Kusters R, van der Weegen W (2018). Assessing the Efficacy of an Educational Smartphone or Tablet App With Subdivided and Interactive Content to Increase Patients' Medical Knowledge: Randomized Controlled Trial. JMIR Mhealth Uhealth.

[41] Schneider BC, Schröder J, Berger T, Hohagen F, Meyer B, Späth C, Greiner W, Hautzinger M, Lutz W, Rose M, Vettorazzi E, Moritz S, Klein JP (2018). Bridging the “digital divide”: A comparison of use and effectiveness of an online intervention for depression between Baby Boomers and Millennials. J Affect Disord.

[42] Cabacungan AN, Diamantidis CJ, St Clair Russell J, Strigo TS, Pounds I, Alkon A, Riley JA, Falkovic M, Pendergast JF, Davenport CA, Ellis MJ, Sudan DL, Hill-Briggs F, Browne T, Ephraim PL, Boulware LE (2019). Development of a Telehealth Intervention to Improve Access to Live Donor Kidney Transplantation. Transplant Proc.

[43] Narva AS, Romancito G, Faber T, Steele ME, Kempner KM (2017). Managing CKD by Telemedicine: The Zuni Telenephrology Clinic. Adv Chronic Kidney Dis.

[44] Greenberg AJ, Haney D, Blake KD, Moser RP, Hesse BW (2017). Differences in Access to and Use of Electronic Personal Health Information Between Rural and Urban Residents in the United States. J Rural Health.

[45] Bagchi A, Melamed B, Yeniyurt S, Holzemer W, Reyes D (2018). Telemedicine Delivery for Urban Seniors with Low Computer Literacy: A Pilot Study. Online Journal of Nursing Informatics.

[46] Faulkner L (2003). Beyond the five-user assumption: benefits of increased sample sizes in usability testing. Behav Res Methods Instrum Comput.

[47] (2006). Bailey B.

[48] Fink JC, Doerfler RM, Yoffe MR, Diamantidis CJ, Blumenthal JB, Siddiqui T, Gardner JF, Snitker S, Zhan M (2016). Patient-Reported Safety Events in Chronic Kidney Disease Recorded With an Interactive Voice-Inquiry Dial-Response System: Monthly Report Analysis. J Med Internet Res.

