# Supplementary material for: A Patient Safety Educational Tool for Patients With Chronic Kidney Disease: Development and Usability Study
Source: JMIR Form Res. 2020 May 28;4(5):e16137. doi: 10.2196/16137 (PMC7290458; doi:10.2196/16137)
Supplement: Multimedia Appendix 1 [file formative_v4i5e16137_app1.pdf]

In addition to drinking more fluids, which medicines should Mr. Smith **STOP** taking until his diarrhea and fever go away?

Check all that apply

- ❑ INSULIN-for sugar diabetes
- ❑ FUROSEMIDE (LASIX)- a water pill for blood pressure
- ❑ VITAMIN D- for bones
- ❑ LISINOPRIL- for blood pressure
- ❑ ALLOPURINOL- for gout
- ❑ He should **CONTINUE TO TAKE ALL** of his medications
- ❑ He should **STOP TAKING ALL** of his medications
- ❑ I Don't Know

Medical Problems

- **Diabetes (sugar)**
- **High blood pressure**
- **Acid reflux**
- **Chronic kidney disease (weak kidneys)**

Medications

- **LISINOPRIL- for blood pressure**
- **FUROSEMIDE (LASIX)- a water pill for blood pressure**
- **INSULIN- for sugar diabetes**
- **CALCIUM- for bones**
- **OMPRAZOLE (PRILOSEC)- for acid reflux**

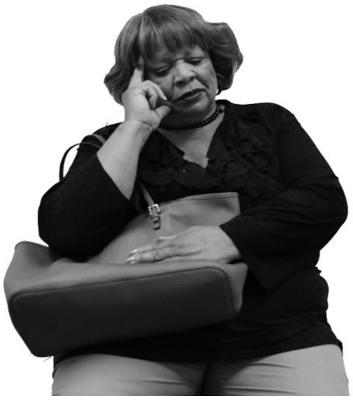

Which one of these blood sugar readings is **MOST DANGEROUS** for Mrs. Johnson?

(Skip if non-diabetic)

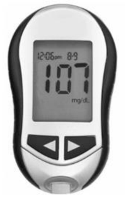

○

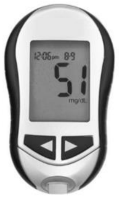

○

I Don't Know

○

Did you know?...

- Many tests are safe for people with weak kidneys
- Some **dyes** or **contrasts** used in tests may make your kidneys sick
- It is important to let **EACH PROVIDER** know about your weak kidneys

It's OK to say something!
