# Supplementary material for: A Patient Safety Educational Tool for Patients With Chronic Kidney Disease: Development and Usability Study
Source: JMIR Form Res. 2020 May 28;4(5):e16137. doi: 10.2196/16137 (PMC7290458; doi:10.2196/16137)
Supplement: Multimedia Appendix 2 [file formative_v4i5e16137_app2.pdf]

SLIDE 2
Welcome! Before we get started, we would like to learn a little bit about you.

Please answer the next few questions as best you can. When you are done with each question, click on the arrow at the bottom of the screen to continue.

Please note, you will not be able to return to earlier questions once you continue onto the next page.

SLIDE 3
Have you ever been told that you have a problem with your kidneys?

When you are done answering the question, click on the arrow at the bottom of the screen to continue.

SLIDE 4
Who first told you about your kidney problems?

When you are done answering the question, click on the arrow at the bottom of the screen to continue.

SLIDE 5
Have you ever seen a kidney doctor?

When you are done answering the question, click on the arrow at the bottom of the screen to continue.

SLIDE 6
How well are your kidneys working? Please answer the question as best you can.

When you are done, click on the arrow at the bottom of the screen to continue.

SLIDE 7
Has anyone ever told you that you have sugar diabetes?

Please answer the question as best you can. When you are done, click on the arrow at the bottom of the screen to continue.

SLIDE 8
How concerned are you about the safety of the medicines you take?

Please answer the question as best you can. When you are done, click on the arrow at the bottom of the screen to continue.

SLIDE 9
How likely do you think it is that the medicines you take are unsafe?

Please answer the question as best you can. When you are done, click on the arrow at the bottom of the screen to continue.

SLIDE 10
Thank you for answering those questions. Now we are going to meet a couple of people and hear about what they can do to keep their kidneys safe.

Meet Mr. Smith. Mr. Smith is a 51 year old store manager. He has high blood pressure, sugar diabetes, gout, and weak kidneys, also known as chronic kidney disease.

Every day, Mr. Smith takes the following medications. Insulin, which is a medicine for sugar diabetes; furosemide, also known as Lasix, which is a diuretic or water pill that helps with his blood pressure; vitamin D, which is a vitamin that helps to keep his bones healthy; Lisinopril, which is a blood pressure medicine; and Allopurinol, which is a medicine that helps with his gout.

Last evening after work, Mr. Smith's belly began to hurt. After a few hours, his pain was no better. He decided to try an over-the-counter pain medicine.

Click on the arrow at the bottom of the screen to continue.

SLIDE 11
Here are some common over-the-counter pain medicines. Are these over-the-counter pain medicines safe or unsafe for Mr. Smith to take for his belly pain?

Please touch the answer below each medicine to show if you think the medicine is safe or unsafe. If you are not sure, you can select "I Don't Know." Answer each as best you can.

When you are done, click on the "CHECK MY ANSWERS button.

SLIDE 12
Many medicines at your local drug store or grocery store do not need to be ordered by a doctor, and can be bought without a prescription. These are called over-the-counter medicines, sometimes called OTC for short.

People who have weak kidneys or chronic kidney disease should not take some of these medicines as they may be harmful to your kidneys. If you have weak kidneys, you should not take a certain type of medicine called a Non-Steroidal Anti-Inflammatory Drug, called NSAID for short.

You have probably seen or even taken NSAIDs in your lifetime. Some NSAID medicines you may have heard of include, ibuprofen, also called Motrin or Advil, or naproxen, which is also called Aleve or Naprosyn.

New medicines come on the shelves all the time, so you should always check with your doctor or pharmacist before taking any new medicine, to make sure it is safe for you and your kidneys.

Click on the arrow at the bottom of the screen to continue.

SLIDE 13
Let's go see how Mr. Smith is doing this morning. Mr. Smith was still having belly pain this morning. He was able to take all of his medicine and eat a little breakfast.

About an hour later, Mr. Smith began feeling dizzy and lightheaded. He used his home blood pressure machine to check his blood pressure. It was normal.

Click on the arrow at the bottom of the screen to continue.

SLIDE 14
Take a look at the list of medicine Mr. Smith takes every day. Could any of Mr. Smith's medicines be causing him to feel dizzy or lightheaded?

Please answer the question as best you can. When you are done, click on the "CHECK MY ANSWERS" button to continue.

SLIDE 15
Which of Mr. Smith's medications might be causing him to feel dizzy and lightheaded? You can pick more than one or check all that apply.

Please answer the question as best you can. When you are done, click on the "CHECK MY ANSWERS" button to continue.

SLIDE 16
What do you think Mr. Smith should do? Please answer the question as best you can. Check all of the answers that you think apply.

When you are done, click on the "CHECK MY ANSWERS" button to continue.

SLIDE 17
Many people with sugar diabetes take a medicine called insulin to lower their blood sugars. But LOW blood sugars, usually less than 70, can be very dangerous. Feeling dizzy or lightheaded may mean that your blood sugar is low. If you feel this way, you should check your blood sugar right away.

Sugar diabetes is a very common cause of weak kidneys or chronic kidney disease. Did you know that as your kidneys get weaker, you are at risk of having low blood sugars? This can happen because your kidneys help get rid of insulin from your body.

As your kidneys get weaker, insulin stays in your blood longer. This may cause low blood sugars which can be dangerous.

Let your doctor know if you are having low blood sugars, so that together you can develop a plan to keep your sugars in the safe zone.

Click on the arrow at the bottom of the screen to continue.

SLIDE 18
Later in the day, Mr. Smith's stomach started to feel upset. About an hour later, he started having bad diarrhea. He skipped work and spent the next day at home. He didn't have much of an appetite, so he didn't eat or drink very much. Unfortunately, his diarrhea did not go away.

The next morning, Mr. Smith felt like he had a fever and took his temperature. Sure enough, he did have a fever.

Click on the arrow at the bottom of the screen to continue.

SLIDE 19
Mr. Smith knows he needs to try and drink more fluids while he is sick, but he is not sure if he should still take his regular medicines.

In addition to drinking more fluids, which medicines should Mr. Smith stop taking until his diarrhea and fever go away?

When you are done, click on the "CHECK MY ANSWERS" button to continue.

SLIDE 20
No matter how hard we try, we all get sick sometimes. If you get sick for a short time and you have a fever, diarrhea, or vomiting, you should stop taking certain medicines until the fever, diarrhea or vomiting has gone away.

Medicines called diuretics, or water pills, may cause you to become dehydrated if you take them when you are sick. Common water pills include furosemide, also called Lasix, and hydrochlorothiazide, commonly called HCTZ for short.

Other medicines called ACE inhibitors and angiotensin receptor blockers, can make your kidneys sick if you take them when you are dehydrated. Examples of these medicines include Lisinopril and Losartan.

When you're sick, it is really important to drink liquids, so that you don't get dehydrated. Start drinking liquids as soon as you can. Try and drink a small amount every so often to stay hydrated.

And when should you start taking your medicines again? Once you are able to eat and drink normally and you feel better. For most people, this is in a day or two. If you are not sure when to start or stop your medicines, be sure to talk your doctor.

Click on the arrow at the bottom of the screen to continue.

SLIDE 21

After another few hours, Mr. Smith still felt awful. He decided that it was time to go to the Emergency Room.

After checking over Mr. Smith and taking some blood tests, the doctor in the Emergency Room told Mr. Smith that she wanted to run more tests on his stomach.

Click on the arrow at the bottom of the screen to continue.

SLIDE 22
It is important that the doctor know what health problems Mr. Smith has before she orders a test of his stomach.

It is VERY IMPORTANT for the doctor to know that Mr. Smith has weak kidneys or chronic kidney disease before running certain tests. Which tests may not be safe for Mr. Smith? You can pick more than one. Please answer the question as best you can.

Check all of the answers that you think apply. When you are done, click on the "CHECK MY ANSWERS button.

SLIDE 23
Sometimes, people need special x-rays, or other tests that use dye, also called contrast, to help figure out a problem in their heart, brain or other parts of their body.

People with weak kidneys or chronic kidney disease may need these tests as well, but the dyes or contrast used in these tests may make your kidneys sick.

Do not assume that the doctors know about your kidney problems when they order a test for you. It is very important for YOU to let them know about your weak kidneys.

Many tests are perfectly safe for people with weak kidneys. And sometimes the test with the dye or contrast might still be the best choice for you. Be sure to talk to your doctor about all of your options, in order to keep your kidneys as safe as possible.

Click on the arrow at the bottom of the screen to continue.

SLIDE 24
Thank you for getting to know Mr. Smith. We are happy to report that after a couple days of rest, he is feeling much better!

Click on the arrow at the bottom of the screen to continue.

SLIDE 25
Now, we'd like you to get to know Mrs. Johnson. Mrs. Johnson is 71 years old. She retired from the post office 3 years ago. She has sugar diabetes, high blood pressure, acid reflux, and weak kidneys, also called chronic kidney disease.

Every day, Mrs. Johnson takes the following medicines. Lisinopril, which is a blood pressure medicine; furosemide, also known as Lasix, which is a diuretic or water pill that also helps with her blood pressure; Insulin, which is a medicine for sugar diabetes; Calcium, which helps to keep her bones healthy; and Omeprazole, also called Prilosec, which helps with her acid reflux.

Mrs. Johnson has been helping to take care of her 2 grandchildren for the summer. Earlier in the week, her granddaughter had the stomach flu, and threw up for a couple days. Today, she's back to herself and has been running around the house all day.

Last night, Mrs. Johnson started feeling queasy. By lunch, she was throwing up and felt like she had a fever. She has been trying to drink some water, but is not able to eat much. She doesn't have much appetite.
Click on the arrow at the bottom of the screen to continue.

SLIDE 26
If Mrs. Johnson is still vomiting tomorrow, which of her medicines should she NOT take in the morning? Please answer the question as best you can. Check all of the answers that you think apply.

When you are done, click on the "CHECK MY ANSWERS" button to continue.

SLIDE 27
The next morning, Mrs. Johnson's upset stomach is feeling a little bit better, but she still doesn't feel like eating that much. She woke up feeling lightheaded and dizzy. She decided to check her blood sugar.

Click on the arrow at the bottom of the screen to continue.

SLIDE 28
Which one of these blood sugar readings is MOST DANGEROUS for Mrs. Johnson? Please answer the question as best you can.

When you are done, click on the "CHECK MY ANSWERS" button to continue.

SLIDE 29
Mrs. Johnson was not quite feeling like herself, and she lost her balance. She hit her back on a wooden kitchen chair. Her back was really sore, and she had trouble moving around because of her back pain. She decided to try an over-the-counter pain medicine to see if it might help her feel better.

Click on the arrow at the bottom of the screen to continue.

SLIDE 30
Are these over-the-counter pain medicines safe or unsafe for Mrs. Johnson to take for her back pain? Please answer the questions as best you can.

When you are done, click on the "CHECK MY ANSWERS" button to continue.

SLIDE 31
Later that night, Mrs. Johnson was feeling dizzy and her back was very sore. The next morning, her daughter found out how Mrs. Johnson was feeling, and she insisted on taking her mom to her primary care doctor to be checked out.

After getting some fluids in the doctor's office, Mrs. Johnson's dizziness was better. But, her back still hurt.

Click on the arrow at the bottom of the screen to continue.

SLIDE 32
The doctor decided to order a medical test to get a closer look at Mrs. Johnson's back. Which of the following tests are probably SAFE for Mrs. Johnson to have? Please answer the question as best you can.

When you are done, click on the "CHECK MY ANSWERS" button to continue.

SLIDE 33
We are happy to report that after a few weeks of rest, Mrs. Johnson is feeling much better.

She is back to running after those grandkids again. They never seem to run out of energy!

Click on the arrow at the bottom of the screen to continue.

SLIDE 34
Congratulations! You have reached the end of our program.

We would love to get your feedback! Please continue on to the next page and let us know your thoughts.

SLIDE 35
Please tell us whether you agree or disagree with the following.

Overall, I enjoyed this learning activity.

When you are done answering the question, click on the arrow at the bottom of the screen to continue.

SLIDE 36
The mobile tablet was easy to use.

When you are done answering the question, click on the arrow at the bottom of the screen to continue.

SLIDE 37

After finishing this activity, I know more about how to keep my kidneys safe.

When you are done answering the question, click on the arrow at the bottom of the screen to continue.

SLIDE 38
I would recommend this learning activity to others.

When you are done answering the question, click on the arrow at the bottom of the screen to continue.

SLIDE 39
On a scale of 1 to 9, with 1 being poor and 9 being excellent, how would you rate this activity?

When you are done answering the question, click on the arrow at the bottom of the screen to continue.

SLIDE 40
Thank you again for taking part in this study.

You can now hand the tablet and headphones back to the coordinator.
