# Supplementary material for: A Patient Safety Educational Tool for Patients With Chronic Kidney Disease: Development and Usability Study
Source: JMIR Form Res. 2020 May 28;4(5):e16137. doi: 10.2196/16137 (PMC7290458; doi:10.2196/16137)
Supplement: Multimedia Appendix 3 [file formative_v4i5e16137_app3.pdf]

Task #	Task Description
1.	When you are done with each question, click on the arrow at the bottom of the screen to continue.<br><br>Slide 2: Click arrow at bottom of page
2.	Have you ever been told that you have a problem with your kidneys? Yes or No<br><br>Slide 3: Answer question on page
3.	Slide 3: Click arrow at bottom of page Problems with kidneys
4.	Who first told you about your kidney problems?<br><br>Slide 4: Answer question on page
5.	Slide 4: Click arrow at bottom of page
6.	Have you ever seen a kidney doctor?<br><br>Slide 5: Answer question on page
7.	Slide 5: Click arrow at bottom of page
8.	How well are your kidneys working?<br><br>Slide 6: Answer question on page
9.	Slide 6: Click arrow at bottom of page
10.	Has anyone ever told you that you have sugar diabetes? Yes, No, I don't know<br><br>Slide 7: Answer question on page
11.	Slide 7: Click arrow at bottom of page

12.	How concerned are you about the **SAFETY** of the medicines you take?<br><br>Slide 8: Answer question on page
13.	Slide 8: Click arrow at bottom of page
14.	How likely do you think it is that medicines you take are **UNSAFE**?<br><br>Slide 9: Answer question on page
15.	Slide 9: Click arrow at bottom of page
	Scenario 1
16.	Slide of Mr. Smith’s medical problems and medications<br><br>Slide 10: Click arrow at bottom of page
17.	Are these pain medicines safe or unsafe for Mr. Smith to take?<br><br>Slide 11 : Select answer for the Motrin bottle
18.	Are these pain medicines safe or unsafe for Mr. Smith to take?<br><br>Slide 11: Select answer for the Tylenol bottle
19.	Are these pain medicines safe or unsafe for Mr. Smith to take?<br><br>Slide 11: Select answer for the Ibuprofen bottle
20.	Are these pain medicines safe or unsafe for Mr. Smith to take?<br><br>Slide 11: Select answer for the Aleve bottle

21.	Are these pain medicines safe or unsafe for Mr. Smith to take?<br><br>Slide 11: Select answer for the Advil bottle
22.	Slide 11: Click "CHECK MY ANSWERS" button
23.	Slide 12: Click NEXT
24.	Did you know?...(information about medications)<br><br>Slide 12: Click arrow at bottom of page
25.	Slide of Mr. Smith's medical problems and medications (skipped if non-diabetic)<br><br>Slide 13: Click arrow at bottom of page
26.	Could any of Mr. Smith's medications be causing him to feel dizzy or lightheaded? (skipped if non-diabetic)<br><br>Slide 14: Answer question on page
27.	Which of Mr. Smith's medications might be causing him to feel dizzy and lightheaded? (skipped if non-diabetic)<br><br>Slide 15: Click "CHECK MY ANSWERS" button
28.	(skipped if non-diabetic)<br><br>Slide 15: Click NEXT
29.	What should Mr. Smith do? (Skipped if-nondiabetic)<br><br>Slide 16: Check all answers that apply

30.	(skipped if non-diabetic)<br><br>Slide 16: Click “CHECK MY ANSWERS” button
31.	(skipped if non-diabetic)<br><br>Slide 16: Click NEXT
32.	Did you know?... (information about medications) (Skipped if non-diabetic)<br><br>Slide 17: Click NEXT
33.	Slide of Mr. Smith’s medical problems and medications (skipped if non-diabetic)<br><br>Slide 18: Click NEXT
34.	In addition to drinking more fluids, which medicines should Mr. Smith **STOP** taking until his diarrhea and fever go away? (skipped if non-diabetic)<br><br>Slide 19: “CHECK MY ANSWERS” button
35.	(skipped if non-diabetic)<br><br>Slide 19: Click NEXT
36.	Did you know?... (information about medications)<br><br>Slide 20: Click arrow at bottom of page
37.	Picture<br><br>Slide 21: Click arrow at bottom of page
38.	For which medical tests is it **VERY IMPORTANT** for the doctor to know

	that Mr. Smith has chronic kidney disease (weak kidneys)?<br><br>Slide 22: Check all answers that apply
39.	Slide 22: Click "CHECK MY ANSWERS" button
40.	Did you know?... (information about medications)<br><br>Slide 23: Click NEXT
	Scenario 2
41.	Picture<br><br>Slide 24: Click arrow at bottom of page
42.	Slide of Mrs. Johnson's medical problems and medications<br><br>Slide 25: Click arrow at bottom of page
43.	If Mrs. Johnson is still vomiting tomorrow, which of the following medications should she **NOT** take in the morning?<br><br>Slide 26: Check all answers that apply
44.	Slide 26: Click "CHECK MY ANSWERS" button
45.	Slide 26: Click NEXT
46.	Picture (skipped if non-diabetic)<br><br>Slide 27: Click arrow at bottom of page
47.	Which one of these blood sugar readings is **MOST DANGEROUS** for Mrs. Johnson? (skipped if non-diabetic)<br><br>Slide 28: Check all answers that apply

48.	(skipped if non-diabetic)<br><br>Slide 30: Click “CHECK MY ANSWERS” button
49.	(skipped if non-diabetic)<br><br>Slide 30: Click NEXT
50.	Slide of Mrs. Johnson’s medical problems and medications<br><br>Slide 29: Click arrow at bottom of page
51.	Are these pain medicines safe or unsafe for Mrs. Johnson to take?<br><br>Slide 30: Check all answers that apply
52.	Slide 30: Click “CHECK MY ANSWERS” button
53.	Slide 30: Click NEXT
54.	Slide of Mrs. Johnson’s medical problems and medications<br><br>Slide 31: Click arrow at bottom of page
55.	Which of the following tests are probably **SAFE** for Mrs. Johnson to have?<br><br>Slide 32: Answer question on page
56.	Slide 32: Click “CHECK MY ANSWERS” button
57.	Slide 32: Click NEXT
58.	Picture<br><br>Slide 33: Click arrow at bottom of page

59.	Please tell us whether you agree or disagree with the following.<br><br>Overall, I enjoyed this learning activity.<br><br>Slide 35: Select answer
60.	Slide 35: Click NEXT
61.	The mobile tablet was easy to use.<br><br>Slide 36: Select answer
62.	Slide 36: Click NEXT
63.	After finishing this activity, I know more about how to keep my kidneys safe.<br>Slide 37: Select answer
64.	Slide 37: Click NEXT
65.	I would recommend this learning activity to others.<br><br>Slide 38: Select answer
66.	Slide 37: Click NEXT
67.	On a scale of 1 to 9, with 1 being poor and 9 being excellent, how would you rate this activity?<br><br>Slide 39: Select answer
	Picture<br><br>Slide 40:Complete (No Task)
