# Supplementary material for: A Patient Safety Educational Tool for Patients With Chronic Kidney Disease: Development and Usability Study
Source: JMIR Form Res. 2020 May 28;4(5):e16137. doi: 10.2196/16137 (PMC7290458; doi:10.2196/16137)
Supplement: Multimedia Appendix 4 [file formative_v4i5e16137_app4.pdf]

| Participant Testimonials About Using the App |
| --- |
| "The voice directions & reading slowed me down. Enhance the program if person knows correct answers and meds they don’t have to wait." |
| "It was a good learning experience." |
| "Very helpful." |
| "Very Good. It was very interesting as I learned a lot and got helpful reminders about things that I should be doing. This was very helpful to me because after doing this for a while I forgot what I should be doing regarding medications. This could be very helpful to others like me." |
| "I don't buy brand names like the ones seen in the scenario but if it said "NSAID" vs "non-NSAID" I would know it was unsafe, I just didn't know the brand names. Also, I don't have high blood pressure and I don't take blood pressure medications, so I didn't know what those were but I'm happy to have learned something." |
| "I liked the app because it was very helpful with medications, although, if it had taken just 5 more minutes I think it would have been too long." |
| “The app was very good but some of the names of meds are not the same as some popular ones. The voice says a different name than the text which can make it confusing. Also, it would help if you added the function of the medications along with the name that way it might help people to remember what it is by knowing what it does. People who don't have high blood pressure may not know the names of those meds." |
| "Some of the examples seemed too similar making the answers confusing. It also took too long to complete." |
